# Lack of Correlation of WAIS Digit Span with Clox 1 and the Dementia Rating Scale in MCI

**DOI:** 10.1155/2012/829743

**Published:** 2012-04-05

**Authors:** Jevin Jay Lortie, Ruth Remington, Heather Hoffmann, Thomas B. Shea

**Affiliations:** ^1^Department of Psychology, Knox College Galesburg, IL 61401, USA; ^2^Department of Nursing, UMass Lowell, Lowell, MA 01854, USA; ^3^Department of Biological Sciences, UMass Lowell, Lowell, MA 01854, USA; ^4^Center for Cellular Neurobiology and Neurodegeneration Research, UMass Lowell, Lowell, MA 01854, USA

## Abstract

Individuals with MCI declined in performance over 6 months in the Clock-drawing (Clox 1) and the WAIS Digit Span tests, but not in the Dementia Rating Scale (DRS). Individual performance on Clox 1 and Digit Span did not correlate after 6 months. Performance on the Digit Span Test also did not correlate with the DRS, but performance on Clox 1 correlated with the DRS. Performance in Clox 1 was, therefore, not a predictor of performance in the Digit Span Test. These findings support the use of a test battery containing the Digit Span test to detect and track cognitive decline in MCI.

Mild cognitive impairment (MCI) is a condition characterized by subjective cognitive complaints and objective evidence of cognitive deficits beyond that anticipated according to age and education, yet is accompanied by preservation of functional abilities [1, 2]. As such, individuals with MCI are neither cognitively normal nor are they demented. However, MCI is frequently prodromal to Alzheimer's disease (AD) [2], to the extent that MCI has been referred to as early-stage AD [3], and clinical manifestation of AD has been recently subdivided into the stage of mild cognitive impairment (MCI) and the subsequent stage of dementia [4]. In this regard, improved ability to detect the earliest stages of MCI and AD are essential; probably more people than currently detected would benefit from treatment [5–8]. Multiple nonstandardized factors compound early detection, including the variance inherent in initial screening by a family physician/general practitioner [9], differential diagnosis due to comparison of an individual's performance with different normative groups [10], and the heterogeneous nature of MCI [11]. While early detection of cognitive impairment is a daunting task in general, detection of continued decline may be achievable for individuals with MCI since the cognitive performance of such individuals is already being monitored.

Several studies have demonstrated that the use of multiple tests was superior in identification of MCI than was any single test. Such test batteries include the Mini-Mental State Examination (MMSE) [12], the Royal Clock-Drawing test (Clox 1) [13], the Cognitive Capacity Screening Examination (CCSE), the Cognitive Assessment Battery [14–17], as well as novel tests such as Diagnostic characteristics of the Mini-Cog, and a new 50-point test based on expanding selected MMSE items (MMX) [18]. Unfortunately, even though currently utilized tests may accurately diagnose MCI, they do not always predict of the course or eventual outcome of MCI [14]. Accordingly, improved methods are still required not only for initial detection, but also to monitor subtle cognitive change among populations that may predict a transition from MCI to AD.

We present information herein indicating that three tests (Clox 1, the WAIS Digit Span test, and the Dementia Rating Scale-2) provide differential information on cognitive performance in MCI. Individual performance did not correlate among these tests. Our findings suggest that the WAIS Digit Span test, a sensitive and easily administered test, should be incorporated into a test battery for diagnosis and monitoring of MCI.

Participants included 10 community-dwelling individuals (mixed gender,  72 ± 4.3  years of age with  13.5 ± 3  years of education) with an independent diagnosis of MCI who had been randomly assigned to a placebo group under blind conditions as part of a clinical study of a nutraceutical supplement (to be published). Two of these participants withdrew from the study for reasons unrelated to the study itself. Participants completed the Royal Clock-Drawing test (Clox 1) [13, 16], the WAIS Digit-Span test [19], and the Dementia Rating Scale (DRS-2; Psychological Assessment Resources; http://www4.parinc.com/) at enrollment into the study, and 3 and 6 months later. All tests were administered by the same individual. All procedures were approved by the Knox College IRB.

The deltas of individual performance were compared using paired *t* tests. To determine whether or not there was any correlation among performance on these tests, scores of individuals on each test at 3 and 6 month intervals were plotted as a function of each other (e.g., Clox 1 versus total score of the DRS) and a regression line obtained [16].

The participant pool at baseline obtained 87 ± 5% of the maximum possible score on Clox 1, 71 ± 7% of the maximum possible score on the WAIS Digit-Span test (combined forward and backwards scores; no difference was detected in forward versus backward scores), and 94 ± 3% of the maximum possible score on the total DRS, with a range of 91–98% among the various domains of the DRS.

Participants on average displayed a progressive decline in performance in Clox 1 and Digit Span Test over the following 6 months. The decline was more pronounced after 3 months for Clox 1 than for the Digit Span Test, but a similar level of decline for both tests was observed after 6 months. By contrast, no decline was detected in the DRS over 6 months (Figure 1).

The group performance in Clox 1 and the Digit Span Test displayed similar overall declines over 6 months (Figure 1). However, comparison of the performance of individual participants demonstrated distinct differences (Table 1). Participants 1–3 declined in the Digit Span test, while they either improved or maintained performance on Clox 1. This represents 38% of the total participants. Only a single participant declined in both of these tests, and the remaining 50% declined in Clox 1 while improving or maintaining performance in the Digit Span test. Graphing of performance on the Clox 1 test as a function of performance on the Digit-Span test demonstrated a lack of correlation of these tests (Figure 2). Two participants (1 and 3) that declined in the Digit Span, while improving in Clox 1 also declined in DRS (Table 1). One participant (6) declined in both Clox 1 and DRS (but improved in the Digit Span test). The remaining participants (63% of the total) improved in the DRS while either declining or maintaining their performance on Clox 1. These findings indicate that an individual's performance in Clox 1 was not necessarily a predictor of performance in the Digit Span Test. Individual performance on the Digit Span Test also did not correlate with performance on the DRS at 6 months. By contrast, individual performance on Clox 1 correlated with performance on the DRS after 6 months (Figure 2).

The lack of correlation of the decline observed in the Digit Span test with those of either Clox 1 or the DRS indicates that the WAIS Digit Span Test may provide an indication of decline in cognitive performance not necessarily revealed by either Clox 1 or DRS. It was not anticipated that an individual's performance on Clox 1 and the Digit Span test would not correlate at 6 months, since average performance on both of these tests presented a similar decline at 6 months. Similarly, it was not anticipated that an individual's performance on Clox 1 would correlate with that on the DRS at 6 months, since the average performance on Clox 1 had declined but that on the DRS had not. These findings collectively indicate that different aspects of cognitive decline can be revealed by these tests, and that averages of groups can obscure the presence of such declines for individuals.

One interpretation of this phenomenon is that the Clox 1 test, despite its inherent complexity, is contextual, while the Digit Span test is not. The nature of the Clox 1 test is to determine whether or not an individual can visualize a clock, follow a series of instructions, and execute the “change of set” of the designated time to positioning of the hands on the clock [13]. Mildly impaired individuals, however, may draw a clock essentially from long-term memory rather than as a series of steps, and perhaps only display minor difficulty with spacing of numbers and/or placement of hands. The DRS also incorporates contextual memory and/or performance in each of its domains The Digit Span test, by contrast, has no context and relies entirely on short-term memory. In this regard, providing a context for random digits, such as speaking the series with the cadence of a telephone number with area code, a social security number, or a zip code, might generate improved performance. A similar comparison could be devised using either random letters versus letters that eventually spell out familiar words or groups of words that form a simple sentence. Contextual memory may also influence performance on repeat tests, which could not be achieved with the random nature of the Digit Span test.

The findings presented herein were observed among while we were conducting a multisite, follow-up study of a vitamin/nutraceutical formulation previously demonstrated to maintain or improve cognitive performance prior to and during AD (to be published). This ongoing study encompasses a wide range of cognitive impairment, including individuals with MCI and all stages of AD whether or not they are currently receiving pharmacological interventions or initiate additional interventions during the study. As such, there was no inherent goal to determine which if any of our test instruments was more appropriate for any given stage of cognitive impairment. Rather, we utilized a battery of standard tests that our previous studies suggested would be useful for a range of cognitive impairment, and moreover provide comparative information among clusters of participants displaying various degrees of cognitive impairment. The differences in individual versus group decline in these tests were a serendipitous finding, which we report herein in the event that other investigators may wish to consider incorporating the WAIS Digit Span Test into their own respective test battery for MCI. It is unfortunate that we did not conduct the MMSE at baseline, which would allow us to determine whether or not the Wais Digit Span test correlated with the MMSE. The MMSE has been demonstrated to correlate well with both the Clock-drawing test and the DRS [16, 20].

These findings demonstrate that the Clox 1 test was the most sensitive in detecting cognitive decline, but nevertheless, the Digit Span test and the DRS identified cognitive decline in some participants that was not detected by the Clox 1 test. Our findings support the notion that a battery of tests, which encompass a variety of tasks, may maximize early detection and help monitor cognitive change during the course of MCI [14, 16].

## Figures and Tables

**Figure 1:**
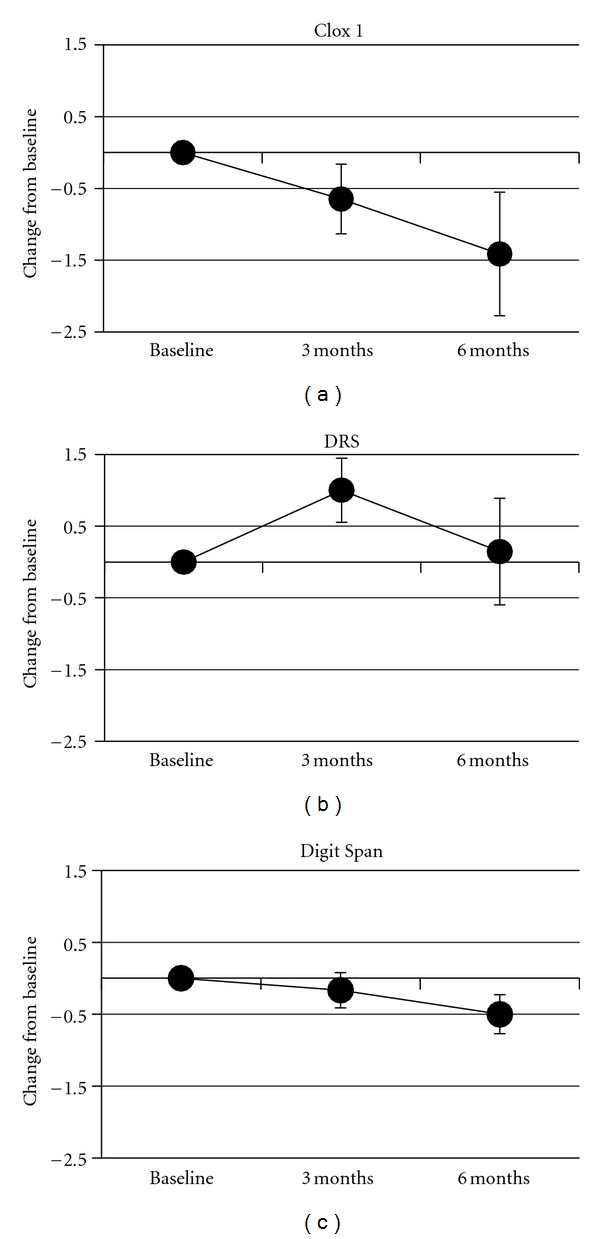
Performance on Clox 1, WAIS Digit Span, and the DRS. The change in value for each individual's performance on each test was calculated at 3- and 6-months. Values represent the mean change (± standard error of the mean). The mean performance at baseline was normalized to 0 for each test to allow comparison among tests. Values for Clox 1 differed statistically from baseline by 3 months. Values for the Digit Span did not differ statistically from baseline until 6 months. Values for the DRS did not differ statistically from baseline at either time point.

**Figure 2:**
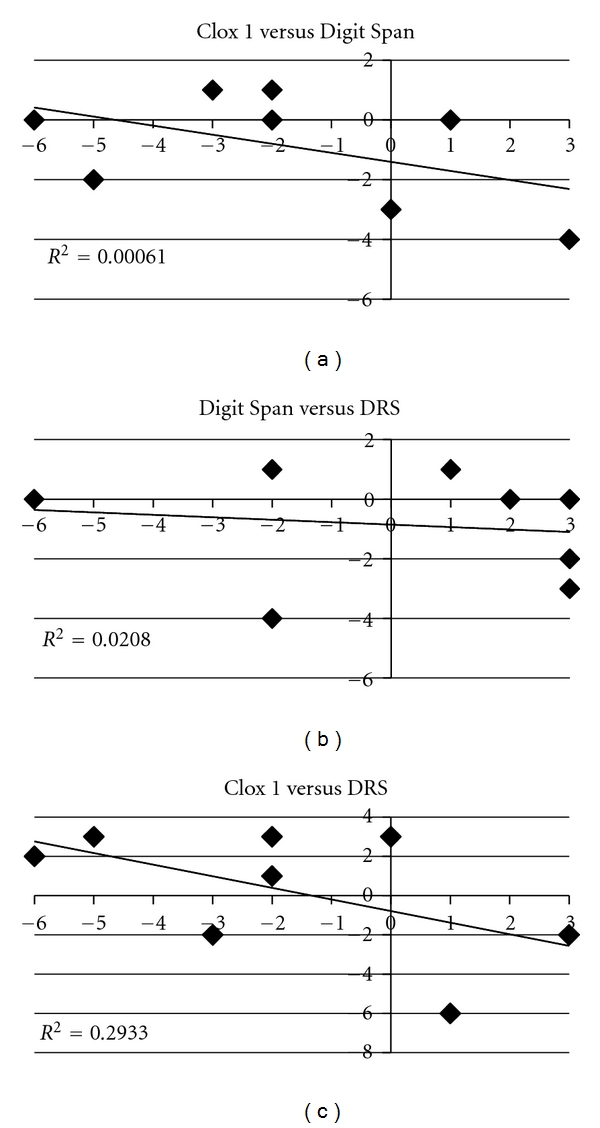
Comparison of individual performance on Clox 1, the WAIS Digit Span and the DRS Each individual's performance on one test was plotted as a function of the other, and a regression line was determined.

**Table 1:** Change of individual performance on Clox 1, WAIS Digit Span, and the DRS. The change in value for each individual's performance on each test over 6 months. Values represent the performance at 6 months minus the respective performance at baseline.

Participant	Clox 1	Digit Span	DRS
1	3	−4	−2
2	0	−3	3
3	1	−2	−6
4	−2	0	3
5	−5	−2	3
6	−3	1	−2
7	−6	0	2
8	−2	1	1
